# Effect of extrusion on the polymerization of wheat glutenin and changes in the gluten network

**DOI:** 10.1007/s13197-020-04413-6

**Published:** 2020-04-18

**Authors:** Feng Jia, Jinshui Wang, Qi Wang, Xia Zhang, Di Chen, Yu Chen, Changfu Zhang

**Affiliations:** College of Biological Engineering, Henan University of Technology, Zhengzhou, 450001 People’s Republic of China

**Keywords:** Glutenin, Extrusion, Polymerization, Gluten network, Microstructure

## Abstract

The changes in the gluten network during extrusion treatment were studied by assessing the polymerization behavior of glutenin. Gluten samples were extruded at different barrel temperatures, screw speeds, and flow rates. The results indicated that high molecular weight glutenin subunits increased while free sulfhydryl groups and low molecular weight glutenin subunits decreased as the screw speeds and flow rates increased during extrusion treatment. Specific β-sheet structures of gluten clearly increased, while α-helices and β-turns fluctuated during extrusion processing, thus forming a tight gluten network. The characteristics of the protein network were evaluated by confocal laser scanning microscopy. The results showed that a homogeneous and denser gluten network was formed at higher extrusion temperatures during the extrusion process, which may be related to the polymerization of low-molecular-weight glutenin subunits. This study provides a theoretical basis for the improvement and regulation of extrusion quality during the gluten extrusion process.

## Introduction

Gluten may include two major types gliadins and glutenins, which are on the basis of their solubility in aqueous alcohol (Wieser 2007). Gliadins are mainly monomeric, but glutenins consist of very large disulfide-linked polymers made up of high molecular weight (HMW) and low molecular weight (LMW) glutenin subunits (Verbauwhede et al. 2019). Glutenins are one of the main major components, which are in charge of imparting strength and elasticity of dough (Yazar et al. 2017), while gliadins confer viscous properties (Bruneel et al. 2010). Although gluten proteins vary from between soft and hard wheat (Jia et al. 2019b), the reactions between gliadin and glutenin have a pivotal role in the formation of wheat gluten network (Li and Gai 2010). Two types of sulfhydryl–disulfide (SH–SS) reactions are crucial during the network formation of gluten. In one, the free-SH groups oxidize into SS bonds (Yue et al. 2019). In the other, there is SH–SS exchange that relates to the reformation or cleavage of SS bonds.

The formation of gluten network is vitally important for many wheat-based food products, like Chinese steam bread (Li and Gai 2010), breads (Wang et al. 2017), and pasta noodles (Yue et al. 2019). The cross-linking of the gluten network is predominantly based on disulfide bonds, although non-disulfide bonds also contribute to the gluten network at higher molding temperatures (Sheng and Huang 2002). The polymerization mechanism of wheat gluten depends on the processing conditions (Zhang and Kong 2014), such as the temperature (Huang et al. 2012), mixing speed (Li and Gai 2010), and extrusion rate (Gao et al. 2017; Li et al. 2017).

The extrusion of wheat gluten may increase the network density, which is observed an increased in elastic modulus and molecular size of gluten (Kucey 1983). The catalytic sites of proteases might be modified and the enzymatic hydrolysis efficiency of wheat gluten could be improved after wheat gluten were treated with extrusion (Hameeda et al. 2008). Heat and a high shear rate, as well as high pressure, cause the mechanical destruction or denaturation of the wheat gluten during extrusion cooking (Bonfil and Posner 2012).

In general, the gluten protein network is developed and set during wheat-based food processing (Delcour et al. 2012). During the processing, the formation of gluten network is now mainly attributed to the oxidation of SH groups of cysteine residues and the formation of SS bonds between molecules or SH–SS interchange reactions (Delcour et al. 2012). Heat-induced gluten aggregation occurs as the result of cross-linking within and between its protein fractions (Delcour et al. 2012). Glutenins could give dough elasticity, as well as gliadins mainly give the dough viscosity and extensibility (Popineau et al. 2020). Glutenin macropolymer (GMP), one of the most important components of glutenin polymer, has been showed to play a pivotal role in the physical properties of the dough and the bread-making quality of wheat flours (Li and Gai 2010). The molecular size distribution of glutenin polymer can be expressed by the content of GMP (Wang et al. 2016).

Little information is so far available on the polymerization behavior of wheat gluten and the changes that occur in the gluten network during extrusion treatment. Variations in the processing conditions during extrusion treatment could affect the expansion capability of gluten during extrusion processing and thus determine the quality of the end products. Clarifying the structural changes in the polymerization of glutenin will provide useful information that will aid efforts to regulate the end-use quality of gluten and to expand the knowledge of the effects of extrusion processing on gluten characteristics. Therefore, the aim of the current investigation was to provide a better understanding of the gluten cross-linking mechanism to develop the theoretical basis for gluten network research on regulating the gluten quality during the extrusion process. Further studies will be performed to discuss the relationship between the extrusion treatment parameters and gluten polymerization.

## Material and methods

### Raw materials

Gluten (brand: FEITIAN, manufactured by Henan Feitian Agricultural Development Co., Ltd., Henan, China) was purchased from a local supermarket. The protein (N × 6.25), moisture, and ash contents were 86.22 ± 0.06%, 7.94 ± 0.01%, and 0.64 ± 0.03%, respectively. A 300 g sample of wheat gluten was added to 100 mL of deionized water and 24 mL of blended oil (peanut oil:soybean oil:rapeseed oil = 2:2:1) and then mixed evenly with a blender.

All other chemicals and reagents used were of analytical grade in this study, and all aqueous solutions were prepared with distilled water. Analyses were completed at least in triplicate.

### Extrusion processing conditions of gluten

A 25 mm co-rotating twin screw extruder (DS32-C, Shandong Sai Xin puffing Machinery Co., Ltd. China) was used for all tests. The ratio of length to diameter of extruder is 20:1, and the length of screw extrusion section is 500 mm. There are three individual heating sections in the extruder settings, with one located at the edge of the barrel, one used as the transition zone to connect the screws and die, and one used for the die. The extruder uses a cylindrical die with a diameter of 3.0 mm to extrude. The screw speed ranged from 40 to 80 rpm, and the flow rate ranged from 30 to 50 g/min. The barrel temperature ranged from 80–140 °C. The extruder ran for 5 min to gain the given sample conditions. Then all collected samples were dried to approximately 5% moisture at 40 °C and stored for 24 h to equilibrate the moisture content. Each sample was analyzed in duplicate.

### Sodium dodecyl sulfate polyacrylamide gel electrophoresis (SDS–PAGE) analysis

Samples (1 g) were dispersed in 10 mL Tris–HCl buffer (0.0625 M, pH 6.8, 2% (w/v) SDS, 10% (v/v) glycerol, and 2% (v/v) 2-mercaptoethanol) and incubated for 2 h. Samples were centrifuged at 8000 × *g* for 10 min after being heated at 100 °C for 5 min. Then, 10 μL of each supernatant was loaded in each lane, and electrophoresis was performed at a constant voltage of 100 V. SDS–PAGE analysis of gluten was carried out using a 12% separating gel (pH 8.8) and 5% stacking gel (pH 6.8) in a vertical electrophoresis cell. The gel was stained with 0.25% (w/v) Coomassie brilliant blue R-250 for 1 h, and then was destained by 7% (v/v) acetic acid. Quantitative analysis of protein gels was determined following a method described by Guo et al. (2018).

### Free –SH group determination

Total contents of free–SH were performed according to the modified method of Beveridge et al. (1974). The formulation of the solvent buffer was 1.2 g EDTA, 6.9 g glycine, and 10.4 g Tris per liter, pH 8.0. First, 200 mg of each sample was suspended in 5.0 mL of 0.05 M sodium phosphate buffer (pH 6.5) containing 1.0 mM tetrasodium ethylenediamine tetraacetate, 3.0 M urea, and 2.0% (w/v) SDS. The mixtures were shaken at ambient temperature for 1 h with vortexing every 10 min. Next, 500 μL 5,5'-dithio-bis (2-nitrobenzoic acid) (DTNB or Ellman’s reagent) reagent was added, after shaking the suspensions gently in the dark for 10 min. The reaction tubes then were centrifuged for 20 min at 1650 × *g*. The volume of the control supernatant was added to 5 mL by solvent buffer for 30 min, and its absorbance was measured. The absorbance of the coloured reaction product was detected at 412 nm against the control (without Ellman’s reagent).

### Fourier transform infrared spectroscopy (FTIR)

KBr discs were prepared in a dry glove box by mixing 1–2 mg of the gluten protein samples with 400 mg KBr and grinding the mixture in a mortar. One hundred milligrams of the mixture was pressed into a 13 mm (diameter) × 1 mm disc. Three discs were made from each sample, and their spectra were recorded on a BioRad FTS 165 FTIR (Varian Limited) spectrometer with a mercury-cadmium-telluride detector. The spectra were gathered over the wavelength range 4000–400 cm^−1^, as well as the spectra were baseline-corrected using an automatic baseline correction method. All samples were analyzed in triplicate.

Then, the software Peak Fit v4.12 was used to peak fit the FTIR spectrum in the wavelength range 1700–1600 cm^−1^. The structural parameters were then calculated. Each spectrum was baseline-corrected according to the method reported by Wellner et al. (2005). Composite bands of the amide I and amide III were determined by using Fourier self-deconvolution with an enhancement factor of K = 1.3 and a bandwidth of 30 cm^−1^ (Herald and Smith 2002).

### GMP isolation

GMP isolation was determined according to the modified method of Don et al. (2003) and Wang et al. (2016). First, the gluten sample (1.0 g) was dispersed in 20 mL of 1.5% (w/v) SDS, followed by centrifugation for 30 min at 10,000 × *g* at 4 °C. The supernatant was discarded, and the gel-like layer on the top of the precipitate was collected as GMP, which was weighed after freeze-drying.

### Confocal laser scanning microscopy analysis

A confocal laser scanning microscope (Model LSM 710, Leica, Germany) was used to observe the morphological characteristics of gluten referring to the modified method Wang et al. (2016). Briefly, pieces of the gluten (0.5 × 0.5 × 0.2 cm) were fixed with a solution of 2.5% (v/v) glutaraldehyde in 0.1 M phosphate buffer of pH 7.0 for 24 h at room temperature and dehydrated with ethanol. Ten-micrometer-thick slices were cut by a rotary microtome (Leica PM2245). The samples were each dyed by solutions of fluorescein isothiocyanate (FITC, 3.5 × 10^−1^ mg/mL) and Rhodamin B (1.3 × 10^−2^ mg/mL), and the dyed samples were washed with deionized water three times to remove the unbonded fluorescent dyes. The dyed samples, before image acquisition, were kept for 4 h in the dark at room temperature. Then, each sample was prepared on a glass slide and observed within 30 min. The samples were measured at an excitation wavelength of 488 nm and emission wavelength of 525 nm.

### Particle size analysis of gluten granules

Particle size analysis of gluten granules was performed according to the modified method of Wang et al. (2016). Gluten granules (10 mg) were ultrasonically dissolved in 10 mL water for 30 min. The particle size distributions of gluten were analyzed with a 633 nm HeNe laser (Nano S90, Malvern Inc., UK) over the range of 1–1000 nm.

### Statistical analysis

Each experiment was done at least in biological triplicate. The statistical analysis was carried out with PASW Statistics 18. Significant differences between the samples were evaluated using one-way analysis of variance (ANOVA) method at the 5% significance level.

## Results and analysis

### Analysis of electrophoretic patterns of wheat gluten

**Fig. 1:**
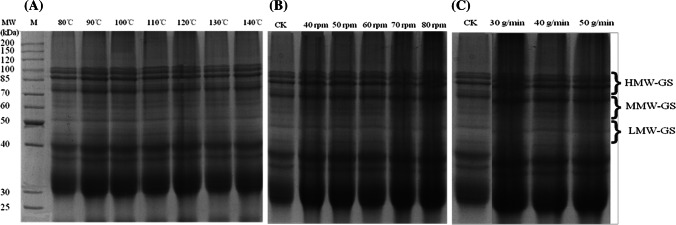
SDS-PAGE electrophoresis analysis of gluten at different extrusion temperatures (**A**), at different screw speeds (**B**), and at different flow rates (**C**)

SDS-PAGE is particularly useful method for identifying individual HMW and LMW glutenin subunits. As shown in Fig. 1, HMW, MMW, and LMW glutenin subunits were separated by SDS-PAGE. The HMW and MMW contents increased slightly as the extrusion temperature increased; however, the LMW content decreased slightly (Fig. 1A). The HMW content increased at higher speeds during the extrusion process (Fig. 1B), and the total LMW content decreased with the increased extrusion flow rate (Fig. 1C). For example, the 102.8 kDa (Fig. 1A) and 60.3 kDa (Fig. 1B) content increased, and the 32.3 kDa content decreased (Fig. 1A). The results indicated that LMW-GS, especially at high extrusion temperatures, might polymerize into HMW-GS during the extrusion.

### Free SH changes induced by the extrusion process

**Fig. 2:**
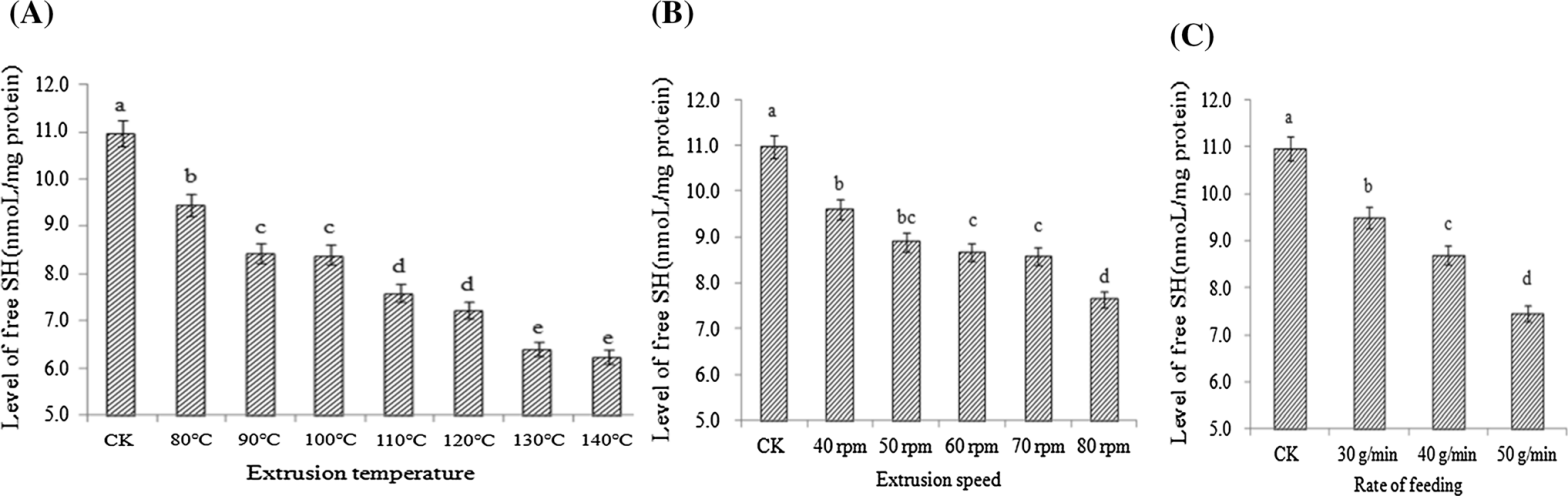
Level (nmol/mg protein) of free SH groups at different extrusion temperatures (**A**), at different screw speeds (**B**), and at different flow rates (**C**)

As shown in Fig. 2A, the SS bond content increased as the extrusion temperature increased, whereas the free SH content decreased. In the same way, the levels of free SH decreased as the screw speed and flow rate increased (Fig. 2B, C). These results suggest that higher extrusion temperatures result in more gluten protein that is polymerized, which may form cross-links through SS bonds.

### Effect of extrusion treatment on the secondary structure of gluten

**Fig. 3:**
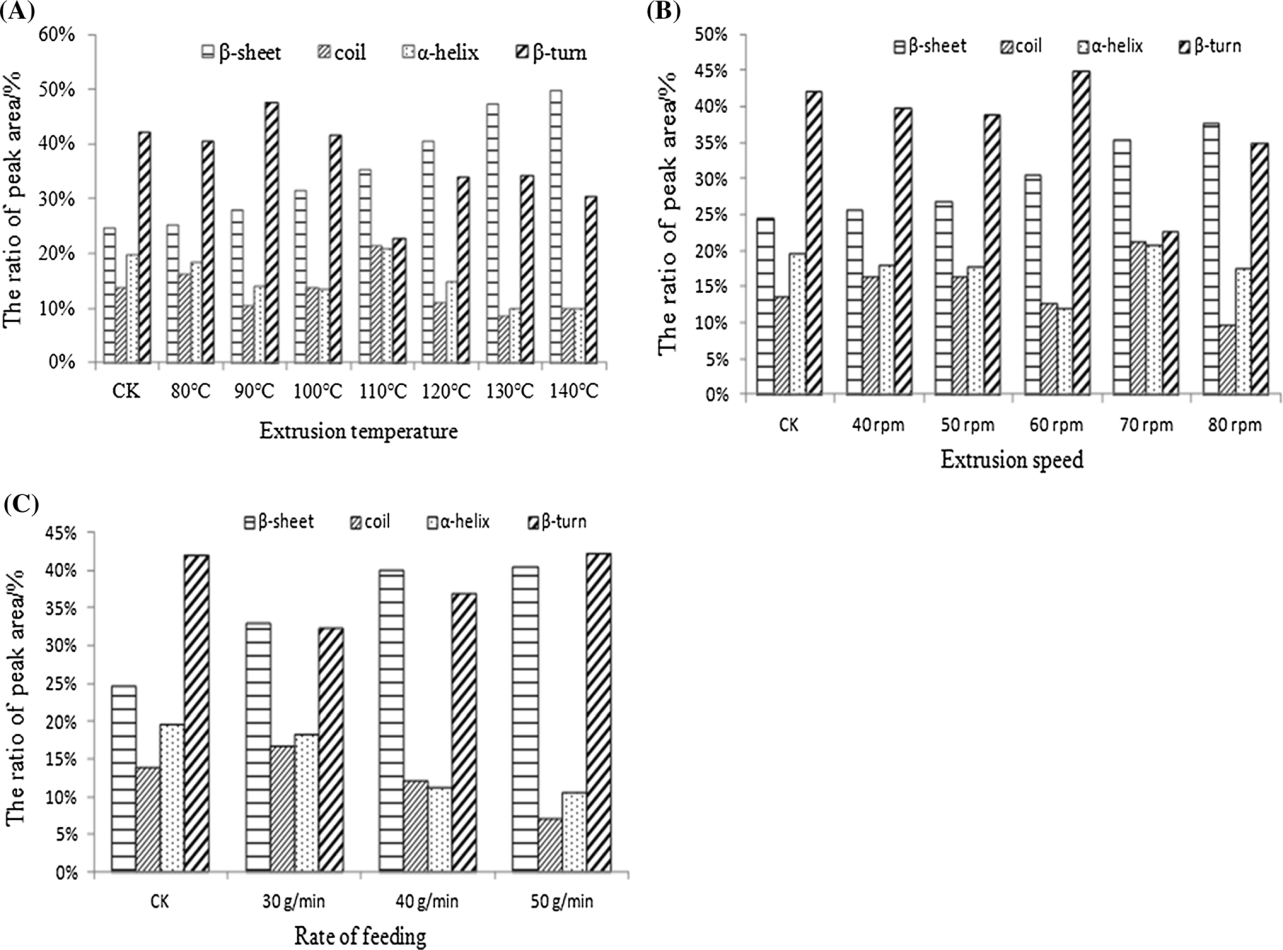
Changes in the gluten secondary structure at different extrusion temperatures (**A**), at different screw speeds (**B**), and at different flow rates (**C**)

The β-sheet content significantly increased, and the α-helix content and β-turn content fluctuated with the barrel temperature increased (Fig. 3A). The β-sheet content clearly increased; however, the α-helix content and the β-turn and coil contents fluctuated with the changes in the screw speed (Fig. 3B). The β-sheet contents increased gradually, while the contents of α-helix and β-turn decreased as the flow rate increased (Fig. 3C). As shown in Fig. 3, four kinds’ secondary structures of protein were detected in all gluten samples, suggesting that the high temperature during extrusion damaged the protein secondary structures, which may have caused the protein to adapt an extended molecular configuration.

### Analysis of GMP contents

**Fig. 4:**
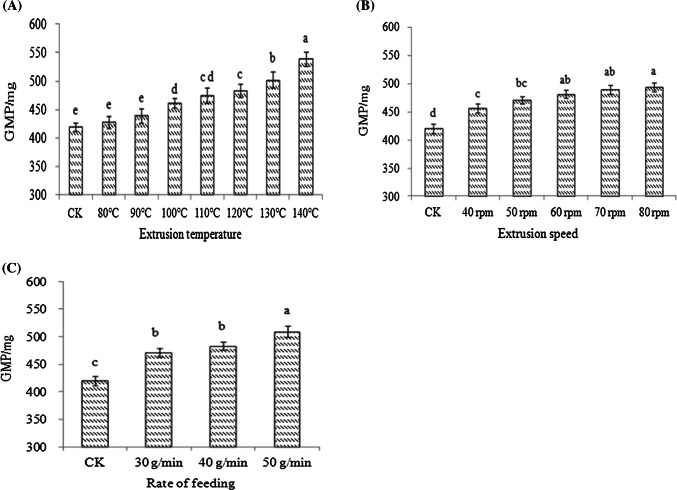
Changes in GMP contents at different extrusion temperatures (**A**), at different screw speeds (**B**), and at different flow rates (**C**)

The GMP contents significantly increased as the extrusion temperature increased (Fig. 4A) because of the decrease in the SDS-extractability of protein during extrusion processing (data not shown). The lowest GMP content was found in the control samples. The GMP contents increased as the screw speed or flow rate increased (Fig. 4B, C), suggesting that the extrusion temperature or pressure may be prompting the polymerization of GMP molecules.

### Confocal laser scanning microscopy

**Fig. 5:**
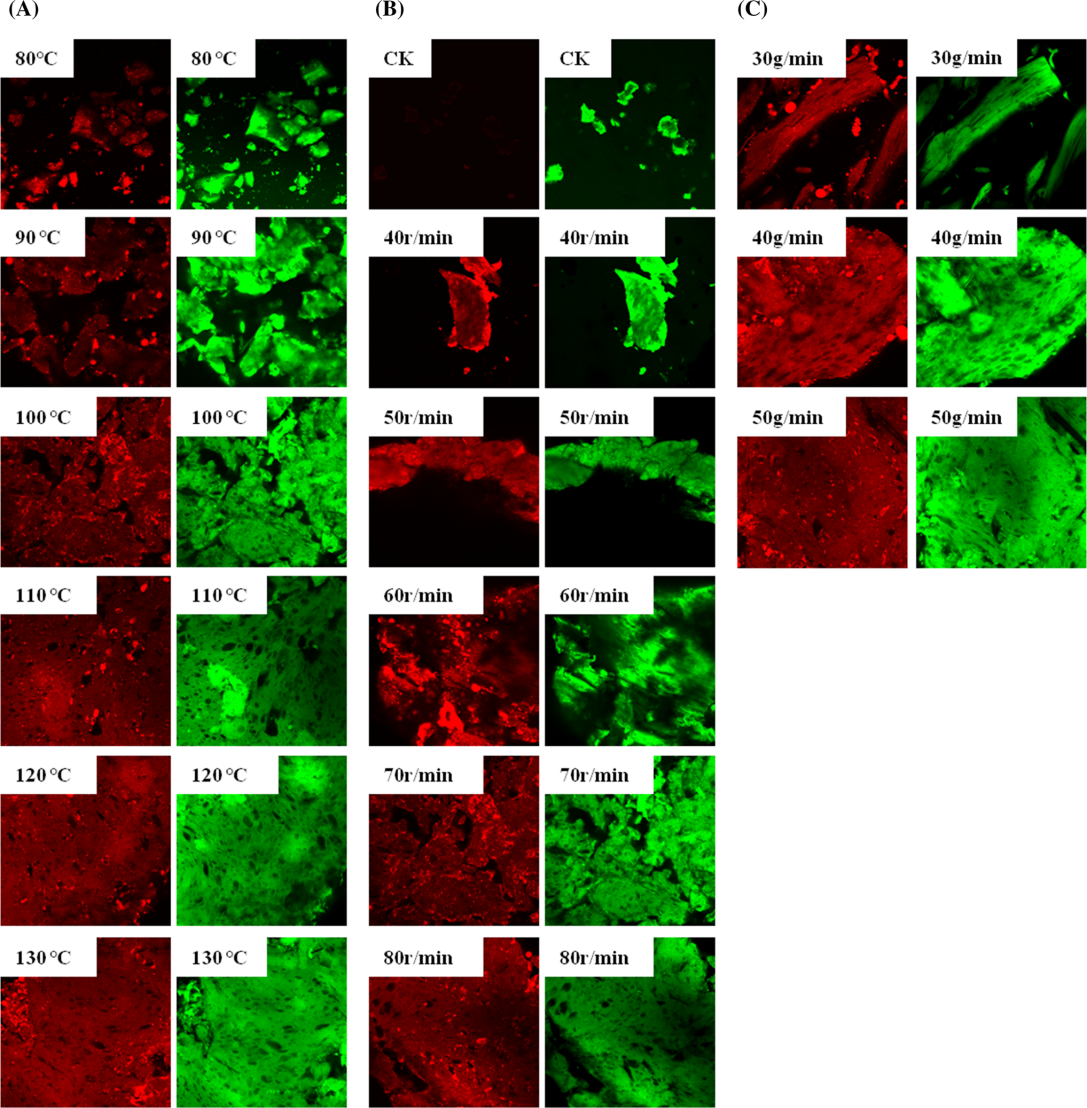
2D elaboration of CLSM images of gluten at different extrusion temperatures (**A**), at different screw speeds (**B**), and at different flow rates (**C**)

When the extruder temperature were of 80 °C, 90 °C, and 100 °C, gluten were observed to form a transient network, and the network morphology of gluten gas cells exhibited apparent distinct characteristics as dark regions at higher extrusion temperatures from 110 to 130 °C (Fig. 5A), suggesting that higher extrusion temperatures led to the formation of a homogeneous protein network, accompanied by a denser gluten network.

As shown in Fig. 5B, the gluten network formed a very dense block and continuous ordered structure with speeds exceeding 70 revolutions per minute. Moreover, the gas cells in the gluten network were clearly not distributed homogenously (Fig. 5B). The CLSM photos show that the gluten network formed a very dense block structure at higher flow rates (Fig. 5C). The structure of the gluten network formed when the flow rate was 40 g/min. Furthermore, the network formation of gluten became denser when the flow rate was 50 g/min. These results suggested that proper flow rates contribute to the network structures of gluten.

### Particle size analysis of gluten

**Fig. 6:**
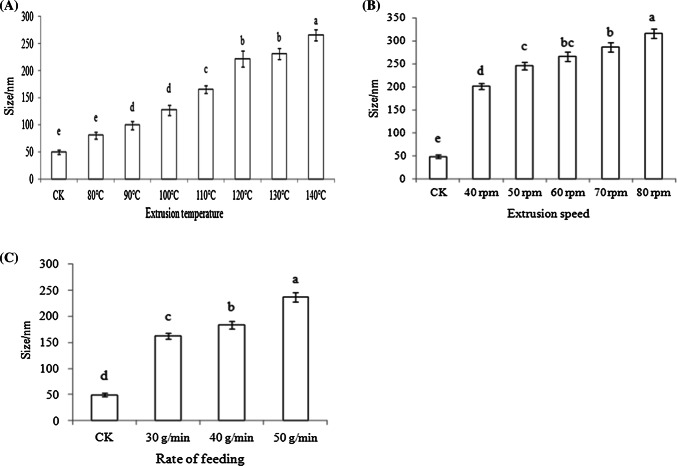
Particle size images of gluten after extrusion at different temperatures (**A**), different screw speeds (**B**), and different rates of feeding (**C**)

The extrusion led to an increase in the gluten particle size as the extrusion temperature increased (Fig. 6A). With the increase of extrusion speed, the particle size of gluten increased gradually (Fig. 6B). With the increase of the rate of feeding, the particle size of gluten also increased gradually (Fig. 6C). The fact that these gluten particle sizes increased as the extrusion temperature, screw speed, or rate of feeding increased suggests that the gluten protein molecules were assembled into a larger structure, possibly because the assembly of gluten led to instability in the gluten network.

## Discussion

Wheat gluten, at least 50 individual components, is a complex heterogeneous mixture of proteins, and there is great variation in the protein components among different genotypes. Glutenin consist of HMW and LMW subunits (Guo et al. 2018). The results of this study show that LMW-GS could polymerize into HMW-GS during the extrusion. This finding is in agreement with that reported by Pietsch et al. (2017), who observed that wheat glutenin polymerization was only influenced by the extruder temperature. The possible reason for this result is that the SS bond content of gluten increased during the extrusion. An increase in SS bonds in proteins would promote the gluten structure formation by protein cross-linking on the dough processing (Guo et al. 2018). The change of disulfide bond content will lead to the agglomeration or depolymerization of proteins. The glutenin depolymerization might lead to instability in the gluten network, providing more possibilities for the rearrangement of protein (Li and Gai 2010). Moreover, the three-dimensional structure of protein network could also be formed during steaming (Li and Gai 2010). High temperature may be the reason to promote the formation of disulfide bonds and the agglomeration of low molecular weight proteins. The present study showed that some LMW were incorporated into the aggregate/combination of HMW by S–S linkage in postharvest wheat maturation (Yue et al. 2019).

FTIR spectrum could be detected the secondary structure of wheat gliadin with the amide I band (1600–1700 cm^−1^) (Mejri et al. 2005; Wang et al. 2020). The percentages of α-helices, β-sheets, and β-turns could be estimated using the ratios of the corresponding area to the total amide I band area. The results showed that the β-sheet content of gluten significantly increased, and the α-helix content and β-turn content fluctuated with the barrel temperature increased during extrusion. The reason for this result is presumably that the change of the secondary structure of proteins content might be related to the reduction of hydrogen bond in gluten. The previous studies have also found that the β-sheets contents of gluten with different oil could significantly increase during extrusion (Jia et al. 2019a). The β-turn content of gluten was related to the flexibility of gluten (Haris and Severcan 1999). However,the β-turn content of gluten decreased during the wheat storage (Jia et al. 2018).

## Conclusion

The SDS-PAGE results showed that the HMW content increased and that the LMW content decreased, as well as the contents of free sulfhydryl groups decreased during gluten extrusion processing. Specific β-sheet structures of gluten clearly increased, while α-helices and β-turns fluctuated during extrusion processing, thus forming a tight gluten network. Moreover, the extrusion contributed to the network formation of gluten at high extrusion temperatures, and the high extrusion temperature could damage the protein secondary structures. Furthermore, CLSM images confirmed that a homogeneous and denser gluten network was formed at the higher extrusion temperatures.
